# Downregulation of MerTK in circulating T cells of patients with non-proliferative diabetic retinopathy

**DOI:** 10.3389/fendo.2024.1509445

**Published:** 2025-01-08

**Authors:** Shimiao Bu, Jiang-Yue Ling, Xiaojun Wu, Liting Zhang, Xiangyu Shi, Lang Huang, Zheng Zhao, Ying Yang, Zongqin Xiang, Yong U. Liu, Yufeng Liu, Yuehong Zhang

**Affiliations:** ^1^ Department of Ophthalmology, The Second Affiliated Hospital, School of Medicine, South China University of Technology, Guangzhou, China; ^2^ Laboratory for Neuroimmunology in Health and Diseases, Center for Medical Research on Innovation and Translation, Institute of Clinical Medicine, The Second Affiliated Hospital, School of Medicine, South China University of Technology, Guangzhou, China; ^3^ Department of Neurology, The First Affiliated Hospital, School of Medicine, Jinan University, Guangzhou, China; ^4^ Department of Neurology, Multi-Omics Research Center for Brain Disorders, The First Affiliated Hospital, University of South China, Hengyang, China

**Keywords:** diabetes mellitus, retinopathy, non-proliferative diabetic retinopathy, PBMC (peripheral blood mononuclear cells), MERTK

## Abstract

**Objective:**

To explore the differential gene expression in peripheral blood immune cells of individuals with type 2 diabetes mellitus (DM), comparing those with and without non-proliferative diabetic retinopathy (NPDR).

**Methods:**

From a pool of 126 potential participants, 60 were selected for detailed analysis. This group included 12 healthy donors (HDs), 22 individuals with DM, and 26 with NPDR. We analyzed peripheral blood mononuclear cells (PBMCs) using RNA sequencing and quantitative PCR (qPCR) to pinpoint differentially expressed genes (DEGs). Western blot and flow cytometry were also employed to evaluate the protein expression of specific genes.

**Results:**

In patients with NPDR compared to those with DM alone, MerTK—a gene implicated in inherited retinal dystrophies due to its mutations—was notably downregulated in PBMCs. Through flow cytometry, we assessed the protein levels and cellular distribution of MerTK, finding a predominant expression in monocytes and myeloid-derived suppressor cells (MDSCs), with a marked reduction in CD4+ and CD8+ T cells, as well as in natural killer T (NKT) cells. Patients with DM demonstrated a significant deviation in the PBMCs composition, particularly in B cells, CD4+ T cells, and NK cells, when compared to HDs.

**Conclusions:**

The study indicates that MerTK expression in T cells within PBMCs could act as a viable blood biomarker for NPDR risk in patients with DM. Furthermore, the regulation of T cells by MerTK might represent a critical pathway through which DM evolves into NPDR.

## Introduction

Diabetic retinopathy (DR), a major ocular complication of diabetes mellitus (DM), is a primary cause of vision loss, especially in individuals over 50 years old (1). Clinically, DR is classified into non-proliferative diabetic retinopathy (NPDR) and advanced proliferative diabetic retinopathy (PDR) (2). NPDR involves vascular basement membrane thickening and microaneurysm formation, progressing to PDR with severe risks due to neovascularization (3). Current DR treatments mainly address symptoms and show varied patient outcomes. Early retinal function and cellular changes preceding visible microangiopathy in DR suggest the necessity for early intervention (4).

Research has shown that individuals with DM and DR experience changes in the composition of immune cells in their bloodstream (5, 6). Bo Li et al. have unveiled a significant association between 35 distinct immune cell phenotypes and the risk of developing DR (7). This alteration in peripheral immune cells is believed to play a crucial role in chronic inflammation, setting the stage for the development and progression of DR (8, 9). This process is not only pivotal in the early stages of NPDR but also in the progression to more severe forms of the condition, such as PDR. PDR is marked by severe complications, including neovascularization, vitreous hemorrhage, and tractional retinal detachment (10). Peripheral inflammation is thought to exacerbate retinopathy by altering the intraocular environment and disrupting the vascular protective mechanisms in the retina, as indicated by Yokomizo et al (11). Thus, targeting the activity of circulating immune cells presents a potential strategy for the early diagnosis and prevention of DR.

Circulating immune cells have systemic effects on inflammation and can penetrate tissues by crossing the parenchyma-blood barriers, occurring during both health and disease states (12). The movement of specific immune cell subsets into and out of tissues is vital for health and disease progression. Particularly in the retina, the interactions between these immune cells and retinal cells can either worsen or alleviate disease-related changes (13–15), highlighting the critical role of peripheral immune regulation in retinopathy’s development and progression. Understanding the mechanisms by which these cells affect retinal diseases is crucial for creating targeted treatments. Such therapies could adjust immune responses to prevent or manage retinopathy effectively, opening new pathways for intervention.

This study seeks to elucidate the pivotal molecular alterations in circulating immune cells that drive the advancement of DM to NPDR. We analyzed peripheral blood mononuclear cells (PBMCs) from patients with and without NPDR to achieve this. Through RNA sequencing (RNA-seq), we discovered a significant reduction in the expression of the Mer tyrosine kinase (MerTK) gene in patients with NPDR, particularly noting its marked decrease in circulating T cells. MerTK, a member of the Tyro/Axl/Mer receptor family, is predominantly expressed on various immune cells such as monocytes/macrophages, dendritic cells, and microglia (16). It plays a vital role in phagocytosis and clearing apoptotic cells, thereby preventing prolonged inflammation. This phagocytic function is critical in maintaining tissue functionality and internal environment stability (17). MerTK has been extensively researched in the context of retinal pigment epithelium and macrophages, where its mutations are linked to severe retinitis pigmentosa and the dysregulation of macrophage functions in phagocytosis and inflammation (18). Therefore, our findings suggest a critical role for MerTK in circulating T cells, potentially influencing the development of DR.

## Methods

### Study subjects

**Figure 1:**
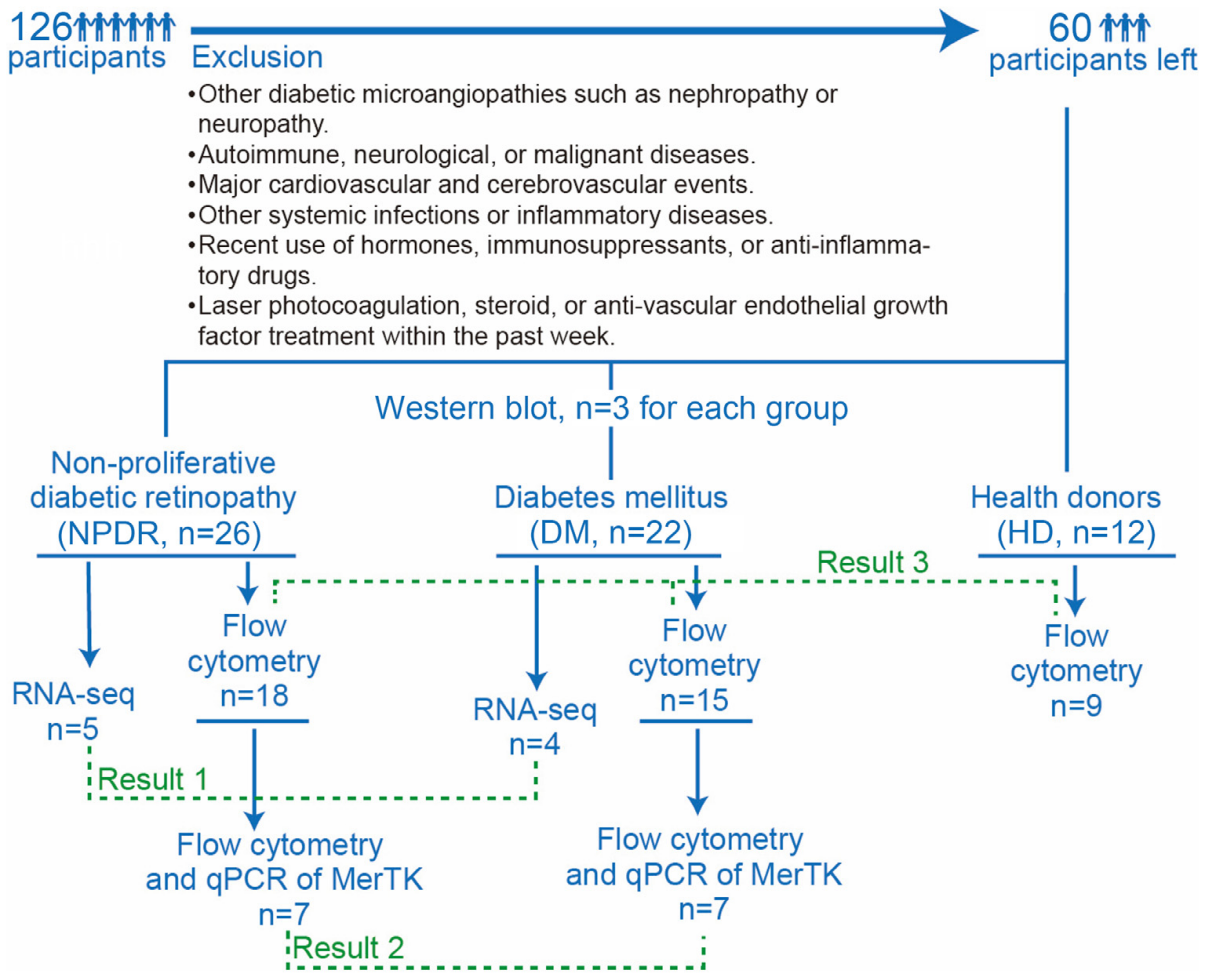
Flow chart of participants enrollment and groups. There were a total of 126 participants, 66 of whom were excluded by exclusion criteria, leaving 60 participants, including 26 in the NPDR group, 22 in the DM group and 12 in the HD group. 5 samples of NPDR and 4 samples of DM were used for RNA-seq. 7 samples of NPDR and 7 samples of DM were used for flow cytometry and qPCR of MerTK. 3 samples of NPDR, 3 samples of DM and 3 samples of HD were used for western blot analysis. 18 samples of NPDR,15 samples of DM and 9 samples of HD were used for flow cytometry.

We enrolled 126 donors from the First People’s Hospital of Guangzhou between 2022 and 2023, and 12 HDs, 22 patients with DM (type 2 diabetes mellitus), and 26 patients with NPDR (type 2 diabetes mellitus with non-proliferative diabetic retinopathy) were selected for analysis (**Figure 1**). Diagnostic criteria adhered to the Clinical Diagnosis and Treatment Criteria for Diabetic Retinopathy in China. Briefly, all participants were subjected to stereoscopic fundus photography for the detection of NPDR utilizing a Non-Mydriatic Fundus Camera through undilated pupils. For every participant, two fundus images centered on the fovea and optic disc for each eye were taken in a darkened room. Two experienced ophthalmologists from the First People’s Hospital of Guangzhou, working independently and in a blinded fashion, assessed each photograph for DR assessment. In cases of discordant evaluations, a third ophthalmologist would make the ultimate decision. The severity of DR in each eye was established, and the subject’s overall classification was determined by the severity observed in the more affected eye.

Inclusion criteria included confirmed DM diagnosis and accurate clinical diagnosis of NPDR grading; diagnosed with type 2 DM; the duration of diabetes ranged from 1 to 20 years; and the participants’ ages ranged from 40 to 90 years. Exclusion criteria encompassed if they were younger than 18 years old or had type 1 diabetes mellitus; other diabetic microangiopathies like nephropathy or neuropathy; autoimmune, neurological, or malignant diseases; major cardiovascular and cerebrovascular events; other systemic infections or inflammatory diseases; recent usage of hormones, immunosuppressants, or anti-inflammatory drugs; and laser photocoagulation, steroid or anti-vascular endothelial growth factor treatment in the past week.

We collected participants’ age, gender, duration of diabetes, and blood pressures. Venous blood samples were drawn after an overnight fast. All biochemical analyses were performed in our hospital, including glycosylated hemoglobin A1c (HbA1c), total cholesterol (TC), triglyceride (TG), high-density lipoprotein cholesterol (HDL), low-density lipoprotein cholesterol (LDL), and estimated glomerular filtration rate (eGFR). This study complied with the Declaration of Helsinki for research involving human subjects and was approved by the research ethics committee of the institute (K-2022-157-01). Informed consent was obtained from all participants per ethical standards. Flow chart of participants enrollment is in **Figure 1**. Detail of participants are in **Supplementary Tables 1**–**4**.

### PBMCs preparation

Peripheral blood (5mL) was collected in EDTA-containing tubes (Sanli, Liuyang, China) and processed within 2 hours. Lymphocyte Separation Medium (LSM, biosharp, Beijing, China) was added to the blood before centrifugation (19). The PBMCs layer was transferred to a new tube, washed with PBS (labgic, Beijing, China), and treated with red blood cell lysis buffer (solarbio, Beijing, China) at room temperature for 5 minutes. After a final PBS wash, the PBMCs were obtained (20).

### Flow cytometry and UMAP analysis

PBMCs were incubated with a mixture of antibodies for 25 minutes at 4°C in the dark. After PBS washing, cells were suspended in 300 uL PBS and analyzed using a BD flow cytometer. FlowJo 10.8.1 software was used for data analysis. Those antibodies used are in **Supplementary Table 5**.

UMAP analysis was conducted using the “UMAP plugin” in FlowJo 10.8.1. High-quality cells were selected with “FlowAI” for gating and doublet discrimination, followed by random selection of 10000 cells per sample using the “Downsample plugin”. Samples were merged into a new FCS file and visualized with UMAP in FlowJo. UMAP outputs were used for “FlowSOM plugin” clustering with nine predefined clusters. “ClusterExplorer plugin” was then used to present the FlowSOM results. Data from the “ClusterExplorer plugin” were normalized in R software for final visualization and cellular annotation accordingly.

### Gating strategies

**Figure 2:**
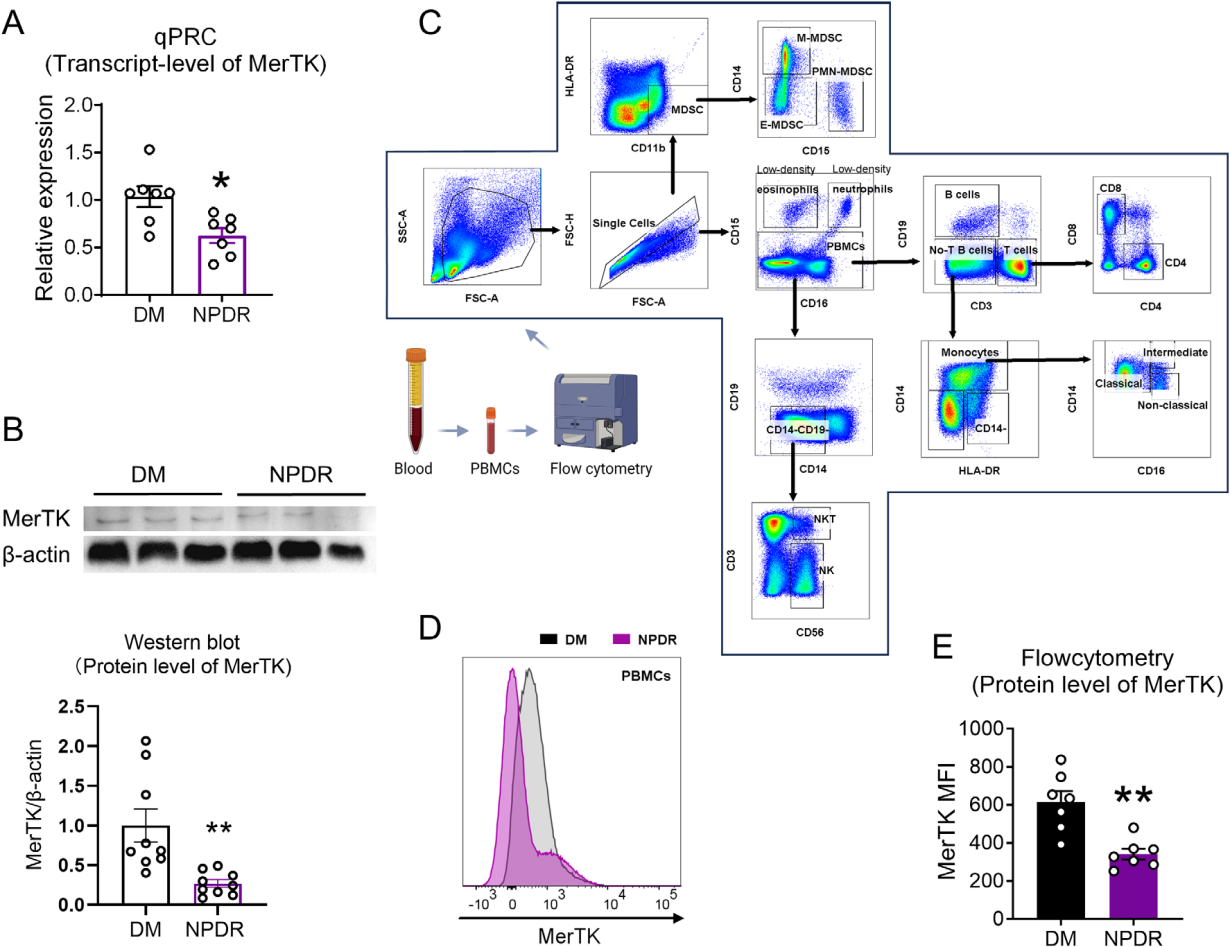
Verification the transcript and protein level of MerTK in NPDR and DM group. **(A)** qPCR verificated the expression level of MerTK in NPDR and DM groups. **(B)** The protein expression of MerTK in PBMCs was measured by western blot analysis, and the ratio of MerTK to β-actin was calculated. n = 3 per group. **(C)** Gating strategies used in flow cytometry analyses to identify subsets of immune cells. Blood sample were isolated into PBMCs and then examined by flow cytometry. Representative image showing forward-scatter area (FSC-A) vs side-scatter area (SSC-A) used to exclude debris/dead cells and the gated population was then plotted as FSC-A vs FSC-H revealing single cells. CD16 vs CD15 showing Low-density eosinophils (CD16-CD15+), Low-density neutrophils (CD16+CD15+) and PBMCs; CD3 vs CD19 showing B cells (CD3-CD19+), No-T B cells (CD3-CD19-) and T cells (CD3+CD19-); CD4 vs CD8 showing CD4+T cells (CD4+CD8-) and CD8+T cells (CD4-CD8+); HLA-DR vs CD14 showing Monocytes (CD14+), then CD14 vs CD16 showing monocyte population comprising of three subsets: Classical monocytes (CD16-CD14+), Intermediate monocytes (CD16+CD14+) and Non-classical monocytes (CD16+CD14-); CD14 vs CD19 showing CD14-CD19- cells, then CD56 VS CD3 showing NK cells (CD56+CD3-) and NKT cells (CD56+CD3+). CD11b VS HLA-DR showing Myeloid-derived suppressor cells (MDSCs, CD11b+HLA-DR-), then CD15 vs CD14 showing MDSCs population comprising of three subsets: E-MDSCs (CD15-CD14-), PMN-MDSCs (CD15+CD14-) and M-MDSCs (CD15-CD14+). **(D)** Representative histograms of MerTK in PBMCs. **(E)** Flow cytometry verificated the expression level of MerTK in NPDR and DM groups. n = 7 per group. The significance of the difference between the results was determined by unpaired T-tests. Data is presented as mean ± SEM, *P<0.05, **P<0.01.

Flow cytometry-acquired cells were gated and classified into various immune cells. Myeloid cells were identified as monocytes (CD14+) and their subpopulations, and lymphoid lineage cells included B cells (CD19+), T cells (CD3+ and its subpopulations), NK cells (CD56+), and NKT cells (CD3+CD56+). The details are in **Figure 2C**.

### Quantitative RT-PCR

RNA was extracted from PBMCs using TRIzol (Invitrogen, Waltham, MA, USA) and quantified spectrophotometrically. cDNA was synthesized from 1μg of RNA using a kit (agbio, Hunan, China). qPCR was performed using SYBR-Green PCR Master Mix (agbio, Hunan, China) on a QuantStudioTM 5 system (Thermo Fisher Scientific, Waltham, MA, USA). MerTK gene expression was normalized to GAPDH and analyzed using the 2-ΔΔCt method (**Table 1**).

**Table 1:** Sequences of MerTK and GAPDH.

MerTK	Forward primer GTACCAGCCTGCCTTGATGT Reverse primer GTGTTTGAAGGCAAGAGGCG
GAPDH	Forward primer AAAGCCTGCCGGTGACTAAC Reverse primer TAGGAAAAGCATCACCCGGAG

### Western blot analysis

PBMCs were lysed on ice for 30 minutes using Western & IP lysis buffer (Beyotime, Jiangsu, China) containing phenylmethanesulfonyl fluoride (Beyotime, Jiangsu, China). Protein extracts (40 μg) were separated on a 10% SDS-PAGE gel and transferred onto polyvinylidene fluoride membranes (Millipore, Massachusetts, USA). Membranes were blocked with 5% non-fat milk at room temperature for 2 hours, then incubated overnight at 4°C with the following primary antibodies: rabbit anti-MerTK (1:1000, Proteintech, Wuhan, China) and mouse anti-β-actin (1:4000, Bioworld, Dublin, USA). After washing, membranes were incubated for 1 hour at room temperature with HRP-conjugated secondary antibodies: goat anti-rabbit IgG (H+L) (1:2000, Proteintech, Wuhan, China) and goat anti-mouse IgG (1:2000, Proteintech, Wuhan, China). Bands were visualized using the SuperSignal West Pico PLUS chemiluminescent substrate (Thermo Fisher Scientific, Waltham, MA, USA) and quantified using ImageJ software, with protein levels normalized to β-actin.

### RNA-seq and analysis

RNA integrity was checked on a 1% agarose gel and quantified with NanoDrop 2000 (Thermo Fisher Scientific, Waltham, MA, USA). Samples with RIN > 7 were sequenced on Illumina HiSeq/Novaseq/MGI2000 instruments (2 x 150 PE). Data were quality-checked, filtered, and aligned to the reference genome.

This study utilized R 4.3.0 for bioinformatics analysis. The expression matrix was derived from raw sequencing data and analyzed using the “deseq2” package in R. The negative binomial regression model was fitted, and hypothesis testing was performed using the Wald test or likelihood ratio test to identify DEGs. Upregulated genes were selected based on the criteria of |log2FC| ≥ 1 and adjusted *P* < 0.05, while downregulated genes were selected based on |log2FC| ≤ -1 and adjusted *P* < 0.05. Volcano plot was generated using the “ggplot2” package, and heatmap was created with the “pheatmap” package. For Gene Ontology (GO) enrichment analysis and Kyoto Encyclopedia of Genes and Genomes (KEGG) pathway enrichment analysis, we used the “ClusterProfiler” package in R software (21).

The raw sequence data reported in this paper have been deposited in the Genome Sequence Archive (Genomics, Proteomics & Bioinformatics 2021) in National Genomics Data Center (Nucleic Acids Res 2021), China National Center for Bioinformation/Beijing Institute of Genomics, Chinese Academy of Sciences (GSA: HRA007945) that are publicly accessible at https://ngdc.cncb.ac.cn/gsa.

### Statistical analyses

Data analysis was conducted using GraphPad Prism 8.0. T-tests and one-way ANOVA were employed for comparisons, with the Mann-Whitney test or Kruskal-Wallis test used when assumptions of normal distribution or homoscedasticity were not met. Mean ± SEM was reported, with *P* < 0.05 considered significant.

## Results

### Downregulation of MerTK in circulating immune cells of patients with NPDR

**Figure 3:**
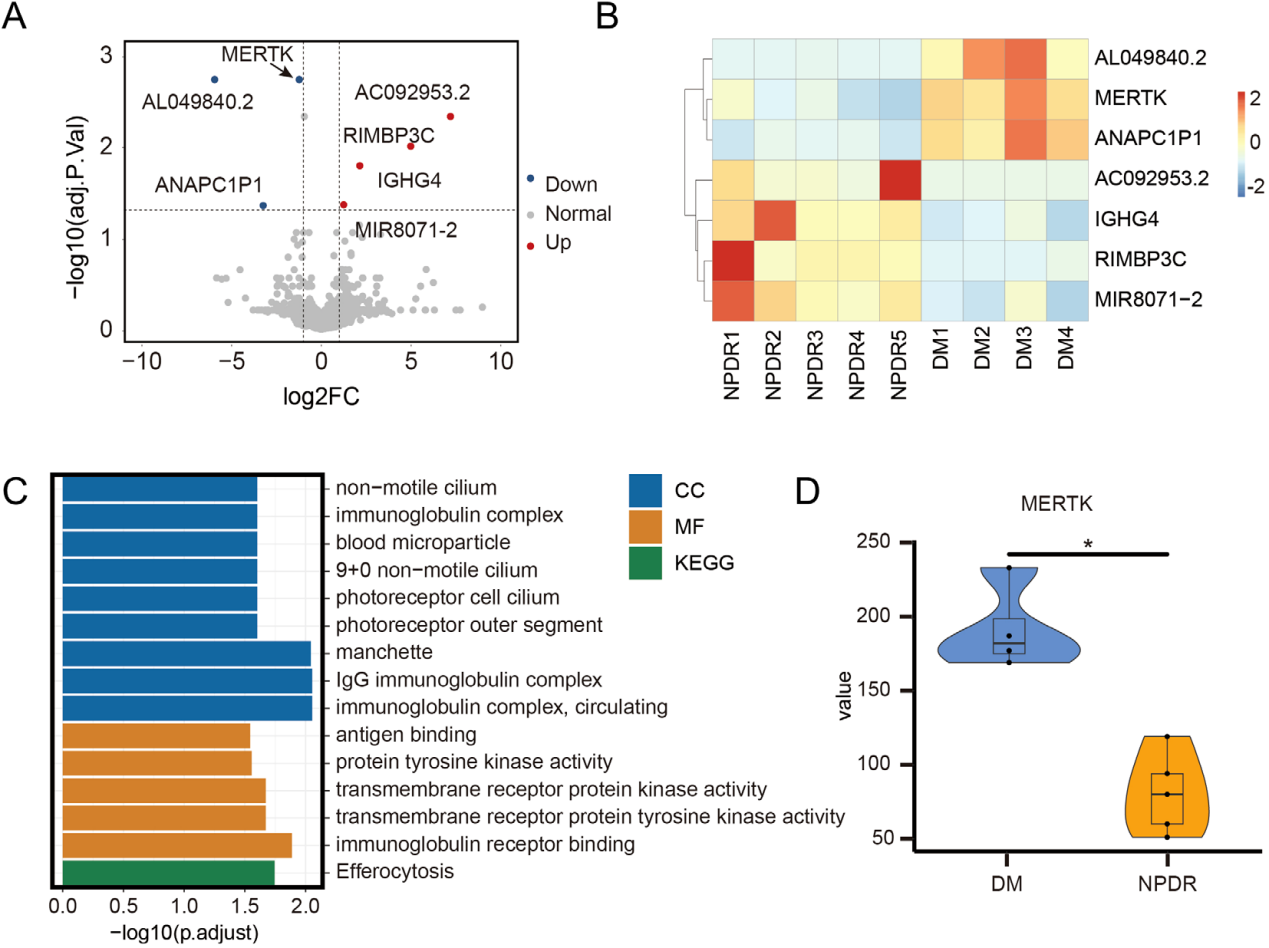
RNA-sequencing reveals the different gens of NPDR compared to DM. The **(A)** volcano plot and **(B)** heatmap of differentially expressed genes between 4 DM and 5 NPDR samples, including 4 upregulated genes and 3 downregulated genes. **(C)** GO and KEGG enrichment analysis of differentially expressed genes. MF: molecular function; CC: cellular component; GO: gene ontology; KEGG: kyoto encyclopedia of genes and genomes. **(D)** Overall MerTK comparison between 4 DM and 5 NPDR samples. The significance of the difference between the results was determined by Mann-Whitney test, *P<0.05.

Following our inclusion and exclusion criteria, we selected the PBMCs of four patients with DM and five patients with NPDR for RNA-seq analysis (**Figure 1**). This analysis revealed seven differentially expressed genes (**Figures 3A, B**), highlighting alterations in biological processes like neutrophil homeostasis and apoptotic cell clearance (**Figure 3C**). Notably, we observed a significant downregulation of MerTK in the NPDR group compared to the DM group (adjusted *P* = 0.02, |log2FC| = -1.232, **Figures 3A, D**). This reduction in MerTK expression at both transcriptional and translational levels was confirmed using qPCR, western blot and flow cytometry (**Figures 2A–E**), indicating a consistent decline of MerTK in PBMCs of patients with NPDR. However, no significant difference in MerTK expression was observed between the HD and DM groups (**Supplementary Figure 1**).

### Specific decline of MerTK expression in T cells of patients with NPDR

**Figure 4:**
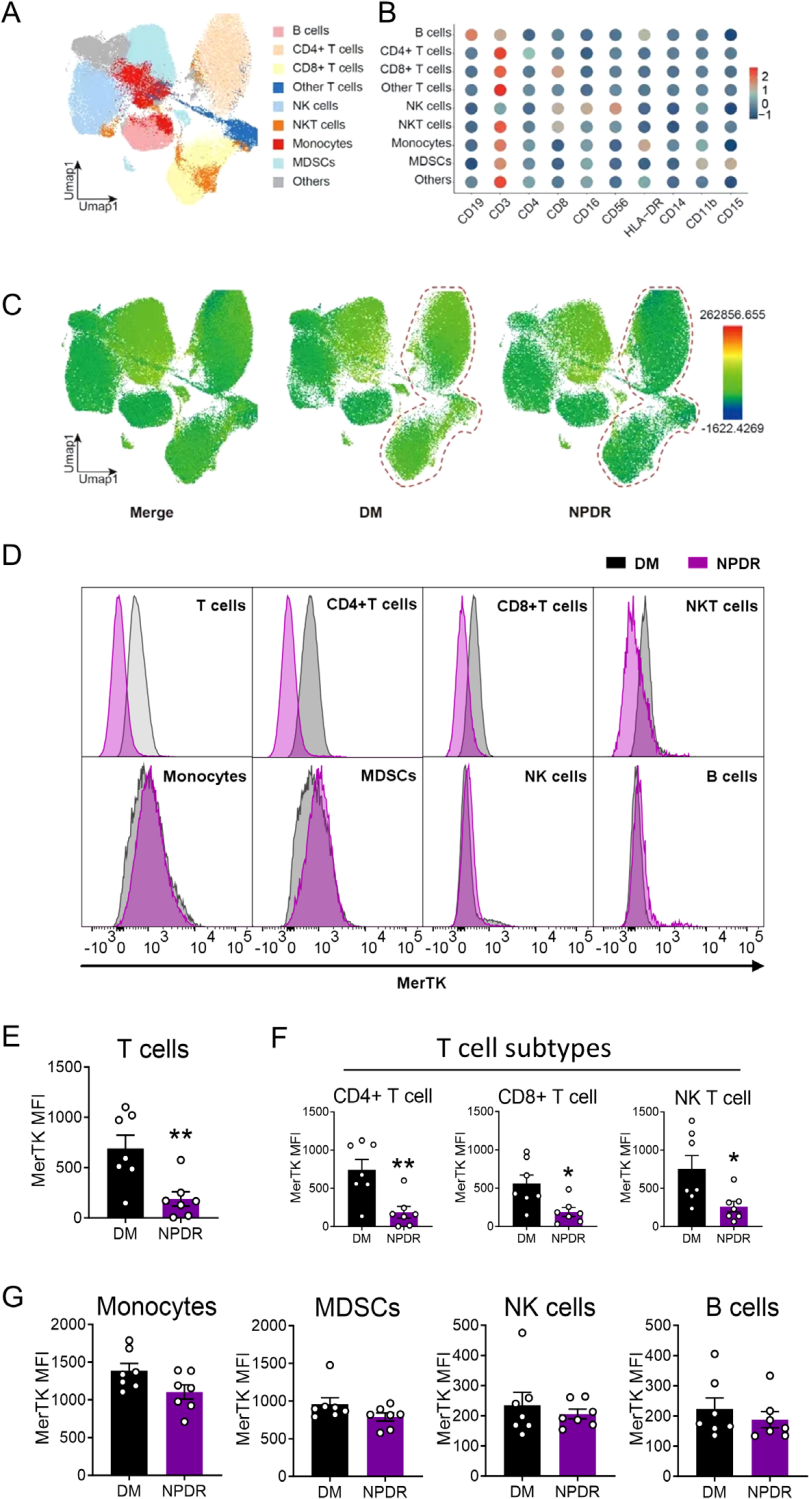
Flow cytometry to demonstrate the expression of MerTK in immune cells. **(A)** UMAP maps of the fourteen patient cohorts (all n = 7) and identified nine clusters. **(B)** Dot plot showing the mean fluorescence intensity (MFI) of clusters. **(C)** UMAP to show the MFI of MerTK in each cluster. **(D)** Representative histograms of MerTK in T cells, CD4+T cells, CD8+T cells, NKT cells, Monocytes, MDSCs, NK cells and B cells. MFI of MerTK in immune cells, including **(E)** T cells and subtypes CD4+ and CD8+T cells, **(F)** NKT cells **(G)** Monocytes, MDSCs, NK cells and B cells. The significance of the difference between the results was determined by unpaired T-tests and Mann-Whitney test. Data are presented as mean ± SEM, *P<0.05; **P<0.01.

Our analysis extended to various immune cell types to investigate MerTK expression differences. Using UMAP analysis, we identified nine distinct immune cell clusters (**Figures 4A, B**). MerTK expression was primarily noted in monocytes and MDSCs, with lesser amounts in NK and NKT cells, and minimal in T cells, including CD4+ and CD8+T cells, and the lowest in B cells (**Figures 4C, D**). In the NPDR group, significant reductions in MerTK expression were observed specifically in CD4+, CD8+, and NK T cells (*P* < 0.01, *P* < 0.05, *P* < 0.05, respectively) (**Figures 4E, F**). Conversely, monocytes, B cells, NK cells, and MDSCs did not show significant expression changes between DM and NPDR groups (**Figure 4G**).

### Immune cell population changes in DM and NPDR

**Figure 5:**
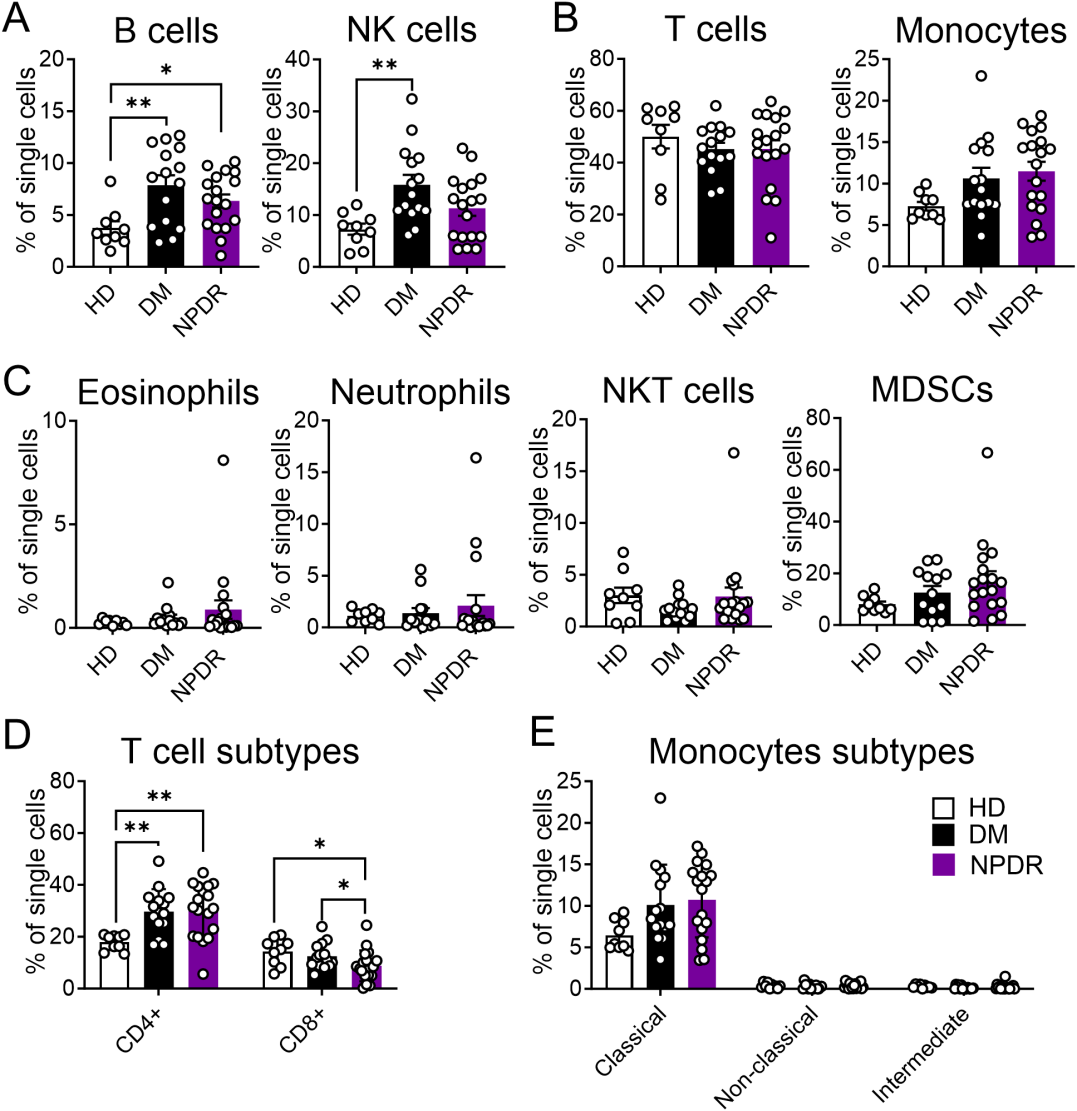
Characterization of immune cells in healthy donors (HD), patients with DM and patients with NPDR. The percentages of total single cells that are: **(A)** B cells and NK cells; **(B)** T cells and Monocytes; **(C)** Low-density eosinophils, Low-density neutrophils, NKT cells and MDSCs; **(D)** T cell subtypes including CD4+T cells and CD8+T cells; **(E)** Monocytes subtypes including Classical monocytes, Non-classical monocytes and Intermediate monocytes. n = 9 for HD, 15 for DM and 18 for NPDR. The significance of the difference between the results was determined by one-way ANOVA and Kruskal-Wallis test. Results are presented as mean ± SEM, *P < 0.05, **P<0.01.

Our study analyzed peripheral blood from 9 HDs, 15 patients with DM (without NPDR), and 18 patients with NPDR. We found significant alterations in the immune cell populations between the DM or NPDR groups and HDs, with no notable differences between DM and NPDR groups. Specifically, B cells increased significantly in both DM and NPDR groups compared to HDs, while NK cells showed a significant increase only in the DM group (**Figure 5A**). T cells, monocytes, low-density eosinophils, low-density neutrophils, NKT cells, and MDSCs did not significantly differ among the three groups (**Figures 5B, C**). Further analysis of T cell and monocyte subtypes revealed an increase only in CD4+T cells in the DM and NPDR groups, with no significant changes in CD8+T cells or in classical, non-classical, and intermediate monocytes (**Figures 5D, E**). These findings suggest that hyperglycemia-induced dysregulation of circulating immune cells may contribute to the onset of NPDR.

## Discussion

MerTK is known for its role in retinal pigment epithelium (RPE), mediating rapid phagocytosis and clearance of photoreceptor debris, with its dysfunction leading to retinal dystrophy and retinitis pigmentosa (22–24). Contrary to its well-documented presence in RPE cells, our study found MerTK to be significantly downregulated in the circulating T cells of patients with NPDR compared to those with DM, particularly in CD4+ and CD8+T cells, as well as NKT cells. Given that T cells are implicated in retinal infiltration during DR, our findings suggest a potential role for MerTK in modulating T cell functions, contributing to the progression from DM to NPDR.

MerTK is a crucial cell surface receptor involved in the innate immune system, involved in the function of a variety of immune cells. One of the most well-known functions of MerTK in macrophages is mediating the clearance of apoptotic cells *in vitro* and vivo (25). Cai et al. found that the signal from MerTK in macrophages can activate the ERK-dependent pathway and inhibit the phosphorylation of 5-lipoxygenase, thus promoting the production of specialized proresolving mediators and promoting the regression of inflammation (26). Meanwhile, TAM family kinases can suppress antitumor immunity and promote resistance to immune-checkpoint inhibitors (27). In dendritic cells, MerTK regulates antigen presentation and immune activation, influencing initial activation and immune tolerance of CD8+T cells (28). The research between MerTK with T cells is also very crucial. Previous research has indicated that MerTK negatively regulates T cell activation (29) and serves as a co-stimulatory receptor on CD8+T cells (30). Studies in pediatric T-cell acute lymphoblastic leukemia showed increased MerTK expression (31), while inhibition of MerTK curtailed T-cell precursor expansion and induced apoptosis (32, 33). These insights bolster our hypothesis that MerTK downregulation could enhance T cell activation and proliferation, intensifying T-cell-mediated immune responses and potentially hastening the transition from DM to DR.

The relationship between DR and DM underscores a complex interplay of factors, including the vitreous environment (11) and systemic inflammation (10, 34) from prolonged hyperglycemia. Our results raise the question of whether changes in circulating T cells mirror alterations within the vitreous environment. The infiltration of these cells through the blood-retina barrier suggests their substantial influence on the vitreous immune response, either protective or harmful (35). Furthermore, the common signaling pathways in blood and retinal T cells hint at a shared mechanism in their regulation.

While the influence of immune cell-mediated inflammation on pathogenesis of DR is widely recognized, there is a lack of detailed quantitative analyses comparing immune cell profiles among patients with DM, those with NPDR, and HD (36). Our study’s comparison of immune cell profiles among HD, DM, and NPDR groups revealed significant changes, particularly in immune cells like B cells, NK cells and T cell subtypes, suggesting their involvement in pathogenesis of DR. Interestingly, we observed increased NK and B cell activities in patients with DM, which might play a role in the early stages of DR (37, 38). The adaptive immune response, represented by CD4+T cell, also showed significant increase, while CD8+ cell showed a decrease, aligning with literature indicating their varied involvement in progression of DR (36). Follicular helper T cells, a subpopulation of CD4+T cells, have also been found to be elevated in patients with DR and diabetic mice, and inhibition of this population attenuates vascular inflammation and neovascularization (39). Some studies have indicated that CD8+T cells can penetrate into retina and concentration significantly higher in vitreous and macular edema in patients with DR, which is associated with poor visual prognosis (40, 41). These can further underline the significance of these cells in DR.

Despite these findings, our study has limitations. We could not fully assess the impact of diabetes medication on PBMCs, with drugs like metformin known to modulate immune responses (42, 43). Additionally, our patient cohort’s demographic skew towards postmenopausal women may influence the observed immune changes. Furthermore, it has been reported that the expression of MerTK in the lymphopoietic system is 59.3% higher in men than in women (44). In our study, although there was no statistical difference in patient gender between the DM and NPDR groups, there were more men in the NPDR group. Therefore, it is necessary to investigate the changes in MerTK expression across different genders. Besides, future research should delve into MerTK’s functional role in T cells and its impact on development of NPDR.

In conclusion, our study elucidates the nuanced differences in immune cell behavior between HD, DM, and NPDR groups, highlighting T cell dysregulation and MerTK downregulation as potential factors in pathogenesis of DR. These findings pave the way for further investigation into the role of immune cells in early development of DR.

## Data Availability

The datasets presented in this study can be found in online repositories. The names of the repository/repositories and accession number(s) can be found in the article/**Supplementary Material**.
