# Supplementary material for: Downregulation of MerTK in circulating T cells of patients with non-proliferative diabetic retinopathy
Source: Front Endocrinol (Lausanne). 2025 Jan 8;15:1509445. doi: 10.3389/fendo.2024.1509445 (PMC11750652; doi:10.3389/fendo.2024.1509445)
Supplement: Supplementary file 1 [file DataSheet1.pdf]

Supplements for:

Downregulation of MerTK in Circulating T cells of Patients with Non-Proliferative Diabetic Retinopathy

Bu et al.

- 1 figure
- 5 tables

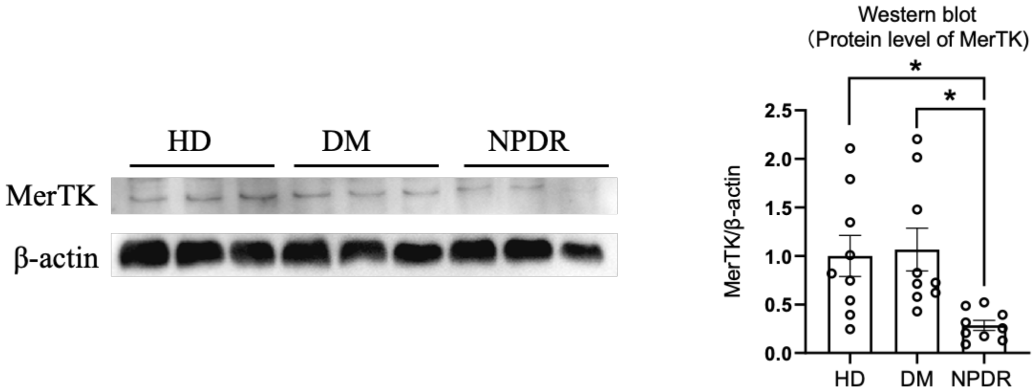

Supplementary Figure 1. Verification the protein level of MerTK in HD, DM and NPDR groups

The protein expression of MerTK from PBMCs was measured by western blot analysis, and calculate the ratio of MerTK to β-actin. n = 3 per group. The significance of the difference between the results was determined by one-way ANOVA. Results are presented as mean ± SEM, *$P < 0.05$.

Supplementary Table 1. Participants‘ general characteristics for the analysis of RNA-seq

Characteristic	DM (n=4)	NPDR (n=5)	*P* value
Age, years	71.50 (3.60)	62.00 (2.92)	0.08
Men, %	25.00	40.00	1.00
Duration of diabetes, years	10.00 (6.25, 10.00)	9.00 (6.5, 10.00)	0.60
T2D, %	100	100	1.00
HbA1c, %	6.7 (6.2, 7.4)	8.0 (6.6, 8.1)	0.26
(mmol/mol)	[50 (44, 58)]	[64 (49, 65)]	
Total cholesterol, mmol/L	5.20 (3.98, 5.37)	5.08 (4.42, 5.25)	0.52
Triglycerides, mmol/L	1.48 (0.24)	1.63 (0.29)	0.71
HDL, mmol/L	1.31 (0.03)	1.23 (0.13)	1.00
LDL, mmol/L	3.00 (0.43)	2.94 (0.21)	0.91
eGFR, mL/min per 1.73m^2	94.67 (9.70)	75.35 (7.04)	0.14
Hypertension, %	100.00	60.00	0.44

The data are summarized as the mean ± SEM for continuous variables with a distribution, the median (interquartile ranges) for continuous variables with a skewed distribution or a numerical proportion for categorical variables.

Supplementary Table 2. Participants‘ general characteristics for the analysis of MerTK

Characteristic	DM (n=7)	NPDR (n=7)	*P* value
Age, years	72.43 (3.24)	63.29 (4.00)	0.10
Men, %	28.57	71.43	0.29
Duration of diabetes, years	10.00 (6.00, 11.00)	10.00 (9.00, 16.00)	0.75
T2D, %	100	100	1.00
HbA1c, %	7.0 (0.1)	8.2 (0.5)	0.07
(mmol/mmol)	[53 (1)]	[66 (5)]	
Total cholesterol, mmol/L	4.61 (0.24)	5.16 (0.79)	0.88
Triglycerides, mmol/L	1.52 (0.78, 1.67)	1.01 (0.72, 3.55)	1.00
HDL, mmol/L	1.23 (0.18)	1.06 (0.13)	0.46
LDL, mmol/L	2.66 (0.26)	2.90 (0.47)	0.65
eGFR, mL/min per $1.73m^2$	79.17 (5.07)	77.61 (12.32)	0.62
Hypertension, %	57.14	28.57	0.59

The data are summarized as the mean ± SEM for continuous variables with a distribution, the median (interquartile ranges) for continuous variables with a skewed distribution or a numerical proportion for categorical variables.

Supplementary Table 3. Participants' general characteristics for the analysis of western blot

Characteristic	HD (n=3)	DM (n=3)	NPDR (n=3)	*P* value
Age, years	68.00 (2.08)	74.00 (7.23)	67.67 (4.33)	0.63
Men, %	33.33	33.33	33.33	1.00
Duration of diabetes, years	NA	7.67 (1.45)	9.33 (0.67)	0.0008
T2D, %	NA	100	100	< 0.0001
HbA1c, %	5.1 (0.1)	7.2 (0.2)	7.5 (0.5)	0.0017
(mmol/mol)	[32 (1)]	[55 (2)]	[58 (5)]	
Total cholesterol, mmol/L	4.83 (0.25)	5.26 (0.59)	5.45 (0.43)	0.63
Triglycerides, mmol/L	1.13 (0.22)	1.42 (0.33)	1.55 (0.40)	0.66
HDL, mmol/L	1.39 (0.08)	1.33 (0.13)	1.30 (0.12)	0.85
LDL, mmol/L	2.78 (0.14)	3.25 (0.17)	2.91 (0.27)	0.29
eGFR, mL/min per 1.73m^2	81.02 (10.09)	76.72 (6.37)	83.78 (11.31)	0.87
Hypertension, %	33.33	33.33	33.33	1.00

The data are summarized as the mean ± SEM for continuous variables with a distribution, the median (interquartile ranges) for continuous variables with a skewed distribution or a numerical proportion for categorical variables.

Supplementary Table 4. Participants' general characteristics for the analysis of immune cells

Characteristic	HD (n=9)	DM (n=15)	NPDR (n=18)	*P* value
Age, years	67.20 (3.89)	69.87 (1.60)	63.00 (2.06)	0.10
Men, %	66.67	33.33	55.56	0.24
Duration of diabetes, years	NA	10.00 (6.00, 12.00)	10.00 (5.75, 15)	< 0.0001
T2D, %	NA	100	100	< 0.0001
HbA1c, % (mmol/mol)	5.5 (0.1) [36 (1)]	7.3 (0.2) [56 (2)]	8.7 (0.4) [71 (4)]	< 0.0001
Total cholesterol, mmol/L	5.03 (0.3)	4.98 (0.27)	5.39 (0.48)	0.70
Triglycerides, mmol/L	0.92 (0.77, 1.39)	1.52 (0.94, 2.40)	1.88 (0.93, 2.84)	0.11
HDL, mmol/L	1.32 (1.27, 1.54)	1.12 (1.01, 1.31)	1.08 (0.74, 1.38)	0.04
LDL, mmol/L	3.12 (0.28)	3.08 (0.27)	3.36 (0.34)	0.78
eGFR, mL/min per 1.73m^2	87.22 (4.93)	79.88 (3.12)	75.52 (8.06)	0.50
Hypertension, %	44.44	46.67	44.44	1.00

The data are summarized as the mean ± SEM for continuous variables with a distribution, the median (interquartile ranges) for continuous variables with a skewed distribution or a numerical proportion for categorical variables.

Supplementary Table 5. Antibodies used for flow cytometry

Antigen	conjugate	dilution	Company
CD3	PE-cy7	1：50	Tonbo
CD11b	PerCP/Cyanine5.5	1：50	Biosciences
CD19	PE-cy5	1：50	Biolegend
CD14	APC	1：50	Biosciences
CD15	BV605	1：50	Biolegend
CD16	BV786	1：50	Biosciences
HLA-DR	APC-cy7	1：50	Tonbo
CD56	PE594	1：50	Biosciences
CD8	BV711	1：50	Biolegend
CD4	FITC	1：50	Biosciences
MerTK	PE	1：50	Biolegend
